# Evaluation after delayed and repeated intervention in the VIPVIZA-extended randomized controlled trial: beneficial results 6 years after baseline

**DOI:** 10.1093/ehjopen/oeag047

**Published:** 2026-04-13

**Authors:** Margareta Norberg, Patrik Wennberg, Per Wester, Anders Själander, Per Liv

**Affiliations:** Department of Public Health and Clinical Medicine, Umeå University, 901 87 Umeå, Sweden; Department of Public Health and Clinical Medicine, Umeå University, 901 87 Umeå, Sweden; Department of Public Health and Clinical Medicine, Umeå University, 901 87 Umeå, Sweden; Department of Public Health and Clinical Medicine, Umeå University, 901 87 Umeå, Sweden; Department of Public Health and Clinical Medicine, Umeå University, 901 87 Umeå, Sweden

**Keywords:** Cardiovascular risk, Cardiovascular risk factors, Primary prevention, Atherosclerosis, Ultrasound imaging, Randomized controlled trial

## Abstract

**Aims:**

The Västerbotten Intervention Programme VIsualiZation of subclinical Atherosclerosis (VIPVIZA) pragmatic randomized controlled trial (RCT) previously reported reduced cardiovascular disease (CVD) risk 3 years after colour-coded information about subclinical atherosclerosis based on carotid ultrasonography and facilitated by nurse-led motivational dialogue. This report evaluated the development of CVD risk and clinical risk factors following the 3-year follow-up, at which point the control group received their first delayed intervention and the intervention group received a repeated VIPVIZA intervention.

**Methods and results:**

Participants (*n* = 3532) were recruited during 2013–2016 and randomized into two groups. Routine primary care managed preventive treatments. At the 6-year follow-up, group differences in CVD risk factors, the European Systematic Coronary Risk Evaluation 2 (SCORE2), and Framingham Risk Score (FRS) were statistically tested. Trajectories of the outcomes in both groups were graphically assessed. The participation rate after 6 years was 75.4%. No significant differences were found between groups in levels of SCORE2, FRS, clinical risk factors, anthropometrics, smoking, or diabetes—except for systolic blood pressure, which was lower in the original intervention group. Risk scores and systolic blood pressure increased in both groups in parallel, while LDL levels decreased and converged. The higher the baseline risk was, the stronger the decrease of LDL cholesterol.

**Conclusion:**

When the delayed VIPVIZA intervention was provided to the control group after 3 years, the beneficial effect appeared similar as previously reported for the intervention group. After 6 years, any differences between groups in CVD risk were no longer seen. Cholesterol levels were greatly reduced in both groups.

**Registration:**

The VIPVIZA trial is registered with www.clinicaltrials.gov (NCT01849575).

## Graphical Abstract

**Graphical Abstract:**
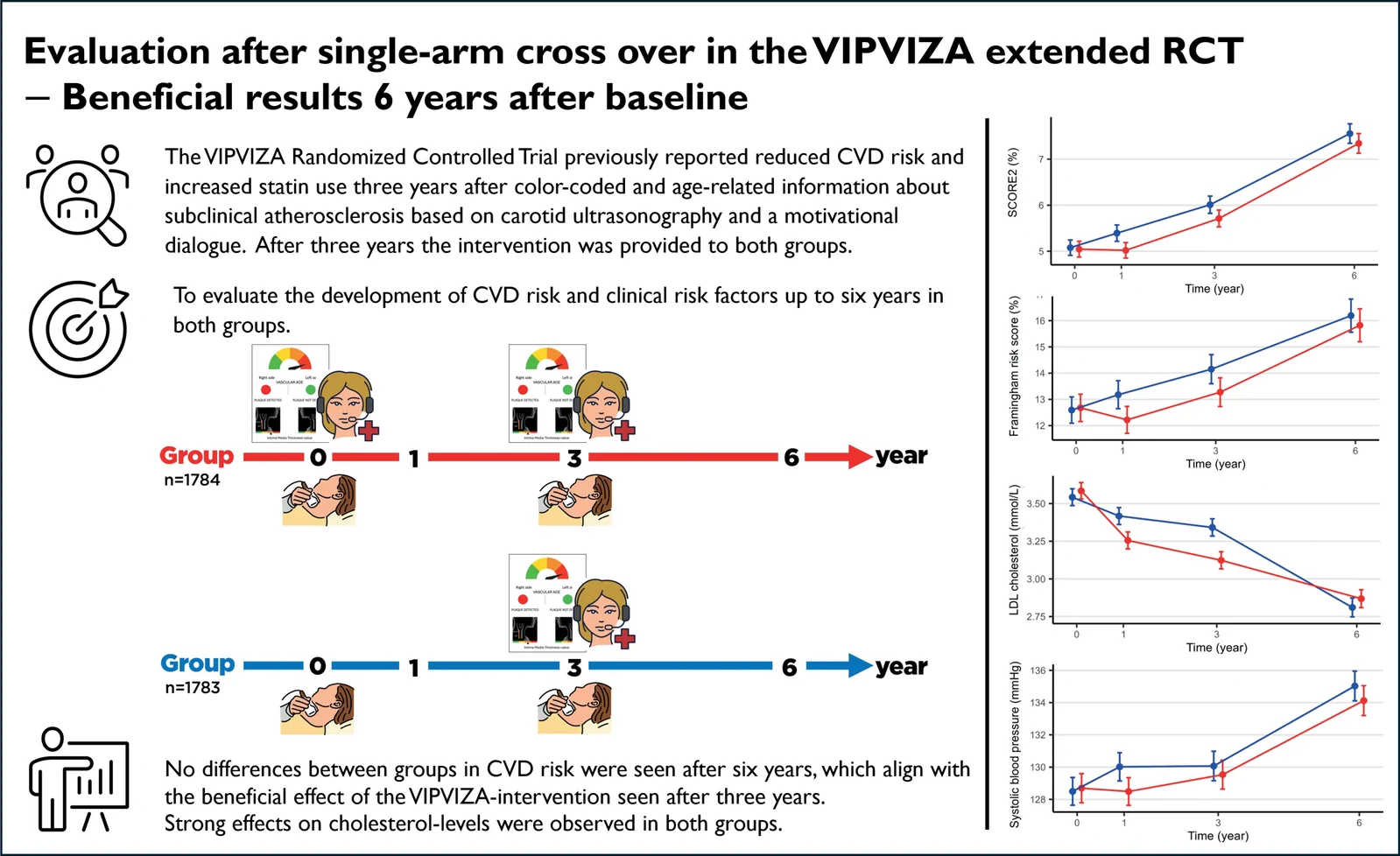

## Introduction

Nonadherence to primary cardiovascular disease (CVD) prevention guidelines still greatly impairs the beneficial effects of preventive measures.^1–3^ To overcome this, current guidelines recommend personalized information to support improved perception of CVD risk, enhanced motivation for lifestyle modification, and optimized adherence to pharmacological treatment.^1^

Visualization of individuals’ health status with imaging techniques enables provision of personalized risk information. In primary CVD prevention strategies, computed tomography (CT) for evaluation of coronary artery calcium (CAC) and vascular ultrasound examinations to visualize subclinical atherosclerosis are usually employed. A systematic review evaluating different CVD risk communication strategies reported in 2022 that provision of personalized visual evidence of an individuals’ health status was effective to promoting primary prevention actions.^4^ This was supported by another systematic review concluding that feedback of individual medical images had the potential to motivate risk-reducing behaviours and reduce CVD risk.^5^ A scoping review found that visualization of individual’s health conditions could have an impact on illness perceptions and medication adherence, particularly when the comprehension of visuals was facilitated by another person, most frequently a health professional or researcher.^6^

However, reviews also point to the risk of bias, the rather low degree of evidence for reported outcome results, and the need for additional adequately powered trials; long-term follow-up studies; and the necessity of developing protocols that elucidate the psychological mechanisms that may link the active intervention components in complex interventions with observed effects on behaviours including medication adherence.^5,6^

This paper reports long-term results from the randomized controlled trial (RCT) Västerbotten Intervention Programme VIsualiZation of subclinical Atherosclerosis (VIPVIZA), assessed 6 years after baseline. VIPVIZA employed imaging of subclinical atherosclerosis based on carotid ultrasound (CUS) examination. One year after baseline, significant differences in Framingham Risk Score (FRS) and the European Coronary Risk Evaluation (SCORE) were reported,^7^ and this effect was sustained up to 3 years.^8^ Also, reduced progression over 3 years of intima–media thickness was reported,^9^ as were increased prescriptions by general practitioners (GPs) of lipid-lowering medication.^10^ At the 3-year follow-up, due to ethical reasons, the result on subclinical atherosclerosis was disclosed to the control group. At that point, both groups received the VIPVIZA intervention, which is based on graphically presenting the extent of subclinical atherosclerosis to each study participant. Thus, the original control group received their first intervention (hereafter referred to as the ‘CI-group’), and the intervention group received a repeated intervention (hereafter referred to as the ‘II-group’). The design is thereafter an extended RCT, still randomized, but without comparator.

This report concerns the long-term development of participants’ CVD risk and major CVD risk factors, with a focus on the period between 3- and 6-year follow-up examinations. Our hypotheses were that the intervention would have a similar impact on the CI-group, and there would be no differences between the two groups at the 6-year follow-up. Since differences in all primary outcomes after 1 and 3 years previously have been published^7,8^ [except the European Systematic Coronary Risk Evaluation 2 (SCORE2), which was introduced in 2021^11^], only the group differences after 6 years were statistically tested. We also graphically present the trajectories of the outcomes from baseline to the 6-year follow-up in both groups.

## Methods

### Design

VIPVIZA is pragmatic parallel-group randomized open blinded end-point trial (PROBE). After the 3 years of follow-up, a delayed intervention for the control group and a repeated intervention for the intervention group were provided.

### Setting

The structure of the VIPVIZA trial during the first 3 years was previously described in detail.^7,8^ Participants in the Västerbotten Intervention Programme (VIP) comprised the recruitment base. The VIP is a population-based CVD prevention programme integrated into ordinary primary care in the county of Västerbotten, Sweden. Since the 1990s, county inhabitants have been invited at ages 40, 50, and 60 years to CVD risk factor screening and an individual motivational dialogue with a nurse aiming at promoting healthy behaviours and preventing CVD. The VIP intervention also includes follow-up and referral to GPs for preventive treatment, according to guidelines.^12^ Only small social selection biases have been observed.^13^ An intention-to-treat (ITT) analysis reported lower standardized total and CV mortality in the target population (VIP participants and nonparticipants combined) compared to the entire Swedish population during 1990–2006.^14^ Thus, the VIPVIZA intervention was provided on top of the VIP intervention. However, results of the VIP examination were concealed to the research team. The planning, conduct, and reporting of the VIPVIZA trial were in accordance with the Helsinki Declaration as revised in 2013. The study was approved by the Regional Ethics Board at Umeå University (Dnr 2011-445-31M, 2012-463-32M, 2013-373-32M) and the Swedish Ethical Review Authority (Dnr 2019-04691). The study is registered at www.clinicaltrials.gov number NCT01849575, where the study protocol and statistical analysis plan (SAP) for the 6-year evaluation are available. The study protocol includes description of patient involvement, details on sample size calculations used for the original trial, and a safety analysis.

### Consent

All participants gave written informed consent before participation in the study.

### Study population and baseline measurements

Participants in VIP who met the inclusion criteria were invited at the occasion of the VIP examination to take part in VIPVIZA, aiming at a study population predominantly comprised of participants at low/moderate risk of CVD. The rationale was that the majority of CVD events occur in this large group. Inclusion criteria were (i) age 40 years and a first-degree relative with CVD before age 60 years, (ii) age 50 years with at least one conventional CVD risk factor, or (iii) age 60 years. Exclusion criteria were significant carotid stenosis (>50% of the lumen), violation of the study protocol, and participation in another CVD prevention trial. Before the inclusion commenced, a statistician generated a randomization list using simple randomization with fixed allocation ratio, to allocate participants consecutively 1:1 to the intervention or the control groups. Availability of the allocation list was kept a minimum and not available for the recruiting primary care nurses. At baseline, 29 April 2013–6 June 2016, all participants underwent a CUS examination to detect subclinical atherosclerosis. One portable CUS machine was used in all examinations, performed by the same team throughout the whole trial, including at healthcare centres in remote rural areas in the county (geographically as large as Switzerland). Intima–media thickness was recorded automatically at prespecified angles, and the presence of plaque was defined in real time by the trained operators who were blinded to randomization status.^9,15,16^ The rationale behind the use of CUS in VIPVIZA was that CT scanning would not be equitable for a population-based trial in a county where a substantial part of the population lives 150–450 km away from the nearest CT scanner. At the same occasion, participants responded to questionnaires on psychosocial and psychological factors relevant to CVD and for behavioural change.

### Intervention in short

The baseline CUS result was used as a communication tool to provide personalized pictorial information about the II-group participant’s current subclinical atherosclerosis and was sent to them. Carotid intima–media thickness was presented with a gauge scoring from green over yellow and orange to red illustrating vascular age from 10 years younger to 10 years older than the chronological age. The presence of plaque was illustrated as a traffic light with a green dot (no plaque) or red dot (plaque) for each side. A stylized picture illustrated the ultrasound image. Written information about the dynamic nature of atherosclerosis, illustrating its potential to slow down or reverse, was included. During a follow-up call 2–4 weeks later, a dialogue based on motivational interviewing with a trained nurse aimed at personalized lifestyle change and to resolve worries and any lack of clarity. Taken together, this low-intensity intervention was intended to improve risk perception and efficacy beliefs and, as a result, improve uptake of preventive measures. The same pictorial and written information was also sent to the II-participant’s primary care physician, along with general information on interpretation of the ultrasound results, but no specific recommendation. During the entire trial, all preventive measures in both groups were provided by primary care. No preventive measures were initiated by the research team, except in case of very high LDL cholesterol levels; when the team checked whether primary care had taken any action, and in cases where the primary healthcare team had not interceded, the participant was prompted to contact primary care.

After 6 months, the same ultrasound result information was again sent to the II-participants, as was general information about a healthy lifestyle. After 9 months and 1.5 years, reminder letters with general lifestyle advice were sent to intervention participants. The intervention components (behavioural change techniques), the ultrasound information, and the written information about atherosclerosis were previously described in detail.^17,18^

### Control

Baseline ultrasound results and written information were disclosed to CI-group participants and their GPs, and no reminders were provided.

### Follow-up examinations

After 1 year, between 8 May 2014 and 10 November 2017, both groups underwent the same risk factor measurements and responded to identical questionnaires about lifestyle, quality of life, and medication as at baseline. All participants and their GPs were informed about risk factor results in a structured template, and depending on risk factor results, general recommendations on lifestyle modification were provided and, when indicated, contact with a GP was prompted.

After 3 years, between 22 August 2016 and 16 June 2019, the same risk factor measurements, questionnaires, and CUS examination as at baseline were repeated. At this time point, as described in the introduction, all participants in both groups and their respective GP received the ultrasound report. This was in accordance with the baseline ethical approval that stipulated that information about subclinical atherosclerosis must not be concealed after this point. At this time point, due to time constraints, the follow-up call was provided to 70% of all participants: all participants in the CI-group and those in the II-group with ‘red results’ for vascular age or the presence of plaque.

This completed the baseline study protocol. However, an additional follow-up after another 3-year period was decided upon. This required a new ethical approval, provision of additional information to the participants, and collection of new informed consent. No more contacts with participants were made between 3- and 6-year follow-ups.

Thus, from 1 December 2019 to 13 May 2023, participants again underwent the same procedures with measurements, questionnaires, and CUS examinations. As in previous waves, this was performed before the provision of any information about the results to participants and their GPs in both groups. In this report, we used data from baseline, 1-year, 3-year, and 6-year follow-up visits.

### Outcomes

Primary outcomes were SCORE2 and FRS. Previous VIPVIZA reports used SCORE,^7,8^ but since SCORE2 was launched in 2021, it was applied in this report instead of SCORE. As prespecified in the SAP, secondary outcomes included total plasma cholesterol, LDL, HDL, and non-HDL cholesterol, plasma triglycerides, systolic and diastolic blood pressure, body mass index (BMI), waist circumference, and smoking status. Additionally, diabetes and body weight were included in the analysis due to their clinical relevance, although not originally specified in the SAP. Diabetes was defined as a self-reported diabetes diagnosis or fasting glucose ≥ 7.0 mmol/L, in accordance with the World Health Organization definition. Smoking was defined as nonsmoker vs. smoking daily or occasionally.

### Statistical analyses

Group differences in continuous outcomes at the 6-year follow-up, with 95% confidence intervals, were estimated using Analysis of Covariance (ANCOVA), adjusted for corresponding baseline measurement. P-triglyceride measurements were log transformed using the natural logarithm prior to analysis. The estimated difference for P-triglycerides was afterwards retransformed using the exponential function and interpreted as a ratio of geometric means. The relative risks of having diabetes or being smoker, comparing II to CI, were estimated using Poisson regression with standard errors from robust Huber–White sandwich estimators.

The primary analyses were performed on complete-case data. Secondary analyses were performed on the ITT population, consisting of all randomized individuals. In the ITT analyses, missing outcome variables, including those on individuals dropping out of the study, were imputed using Multiple Imputation by Chained Equations (MICE) for each of the two groups, separately. Using the R package ‘mice’,^19^ 50 imputed datasets were generated with 30 iterations, with the following variables included as predictors: age, sex, and education, as well as all investigated risk factors measured at both baseline and 6-year follow-up visit. Partial mean matching with 10 potential donors was used for imputing continuous variables, while logistic/multinomial regression was used for categorical variables. A dropout analysis was performed to compare the participants who left the study to those who completed the 6-year follow-up. The trajectory for each of the risk factors across the study time points was visualized with groupwise mean values and prevalences, with 95% confidence intervals, based on the complete-case data. Subgroup analyses were performed across sex, educational level, age groups, and baseline SCORE2 risk levels. The SAP for the 6-year evaluation includes mixed-effects modelling to assess differences in outcomes over time. However, this approach was ultimately not pursued, as the null hypothesis was considered trivially false due to the natural ageing of participants over the course of the study.

The significance level was set at 0.05. In accordance with the prespecified SAP, no correction for multiple comparisons to control the familywise error rate was performed. All analyses were performed using R v4.5.0 (R Core Team, Vienna, Austria).

## Results

**Figure 1:**
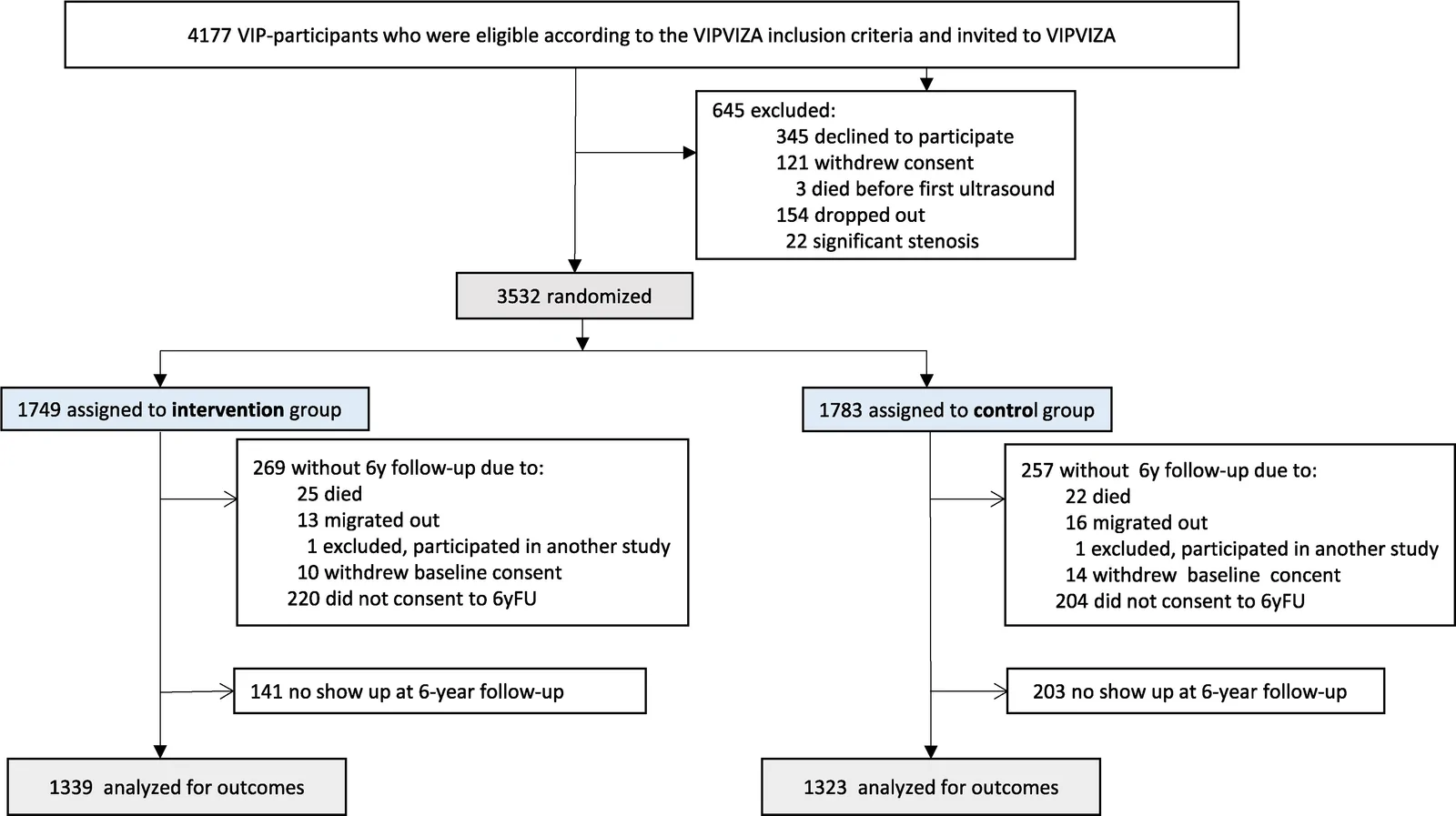
Flow diagram of the VIPVIZA trial showing enrolment and randomization from baseline to the 6-year follow-up visit. VIP, Västerbotten Intervention Programme; VIPVIZA, Västerbotten Intervention Programme VIsualiZation of subclinical Atherosclerosis; II-group, the intervention group that received the first intervention at baseline and a second intervention at the 3-year follow-up; CI-group, the control group that received their first VIPVIZA intervention at the 3-year follow-up.

Out of the 3532 baseline participants, around 90% returned to the 1-year and the 3-year follow-up examinations. Detailed flow charts were previously presented.^7,8^ At the 6-year follow-up, the participation rate was in total 75.4% (*n* = 2662), corresponding to 76.6% (*n* = 1339) and 74.2% (*n* = 1323) in the II-group and the CI-group, respectively (*P* = 0.109). The pattern of reasons for nonparticipation was similar in both groups (*Figure 1*).

The dropout analysis compared baseline levels among participants and nonparticipants in the two groups separately. In the II-group, SCORE2 was higher among dropouts, at 5.4 vs. 5.0 (*P* = 0.026), and diabetes was more prevalent, 8.1% vs. 4.9% (*P* = 0.036). In the CI-group, corresponding values were 5.4 vs. 5.1 (*P* = 0.051) and 7% vs. 5.2% (*P* = 0.190). For all other variables, the pattern was similar in both groups with no differences in distributions of sex and cholesterol levels, while in both groups, dropouts were younger, and a larger proportion had lower educational levels and hypertension, were smokers, and were living alone. Dropouts also had larger waist circumferences, higher fasting glucose levels, and higher blood pressure and triglycerides (see Supplementary material online, *Table S1*).

In this evaluation, 2653 participants are included (47.2% men), since five participants in the II-group and four in the CI-group only participated in the CUS examination and/or responded only to questionnaires on psychosocial factors. Characteristics at the 6-year follow-up are presented in *Table 1*. The levels in risk scores and biomedical risk factors were similar. Variables related to lifestyle showed small nonsignificant differences between the two groups for smoking, weight, BMI, and waist circumference, with lower levels in the II-group. This was true also for biomedical variables (except lipid levels), as well as SCORE2 and FRS. The rate of missing data was <2% in all variables (see Supplementary material online, *Table S2*).

**Table 1:** Characteristics of the VIPVIZA study population at the 6-year follow-up in complete-case analyses

	Male		Female		Total	
	II-group	CI-group	II-group	CI-group	II-group	CI-group
	*n* = 629	*n* = 622	*n* = 707	*n* = 698	*n* = 1334	*n* = 1319
SCORE2	9.2 (3.9)	9.3 (3.8)	5.7 (3.0)	5.9 (2.8)	7.3 (3.9)	7.5 (3.7)
FRS	21.9 (12.1)	22.5 (11.6)	10.3 (7.0)	10.4 (6.3)	15.8 (11.3)	16.1 (11.0)
P-total cholesterol (mmol/L)	4.8 (1.2)	4.8 (1.2)	5.2 (1.2)	5.2 (1.2)	5.1 (1.2)	5.0 (1.2)
P-LDL cholesterol (mmol/L)	2.8 (1.0)	2.8 (1.1)	2.9 (1.1)	2.8 (1.1)	2.9 (1.1)	2.8 (1.1)
P-HDL cholesterol (mmol/L)	1.4 (0.4)	1.4 (0.4)	1.7 (0.4)	1.7 (0.4)	1.6 (0.4)	1.6 (0.4)
P-non-HDL cholesterol (mmol/L)	3.4 (1.2)	3.4 (1.2)	3.4 (2.6, 4.3)	3.3 (2.5, 4.2)	3.5 (1.2)	3.4 (1.2)
P-triglycerides (mmol/L)	1.2 (0.9, 1.7)	1.3 (0.9, 1.8)	1.2 (0.9, 1.6)	1.2 (0.9, 1.6)	1.2 (0.9, 1.7)	1.2 (0.9, 1.7)
Systolic blood pressure (mmHg)	136.0 (15.9)	137.5 (15.2)	132.0 (17.0)	132.5 (16.6)	133.9 (16.6)	134.9 (16.1)
Diastolic blood pressure (mmHg)	86.4 (9.7)	87.0 (9.4)	84.1 (9.4)	83.9 (9.2)	85.2 (9.6)	85.3 (9.4)
Diabetes	73 (11.8%)	78 (12.8%)	56 (8.0%)	60 (8.8%)	129 (9.8%)	138 (10.7%)
BMI (kg/m^2^)	27.8 (4.2)	28.0 (4.6)	27.4 (5.3)	27.9 (5.5)	27.6 (4.8)	27.9 (5.1)
Weight (kg)	88.9 (15.3)	89.7 (16.3)	74.6 (15.1)	75.3 (15.1)	81.3 (16.8)	82.1 (17.2)
Waist (cm)	102.1 (11.7)	103.0 (12.4)	93.4 (13.4)	94.0 (13.2)	97.5 (13.4)	98.3 (13.6)
Smoking	45 (7.2%)	49 (7.9%)	54 (7.7%)	62 (8.9%)	99 (7.4%)	111 (8.4%)
University/academic	186 (29.8%)	186 (30.0%)	295 (42.2%)	297 (43.1%)	481 (36.4%)	483 (36.9%)

Mean levels and SD are shown for continuous variables, except for triglycerides, which are reported as median and interquartile range due to skewness. Categorical variables are presented as number and percentages. Education was registered at baseline.

SCORE2, the European Coronary Risk Evaluation 2; FRS, Framingham Risk Score; BMI, body mass index.

Our main results in complete cases and ITT analyses show that 3 years after the single-arm cross-over, there were no statistically significant differences after adjustment for baseline level, sex, and education, either in SCORE2 or in FRS (*Table 2*). Overall, differences in CVD risk scores, blood pressure levels, and BMI were in favour of the II-group in both analyses. The only statistically significant result was in systolic blood pressure in the ITT analysis (*P* = 0.036), and this was nearly significant in the complete-case analysis (*P* = 0.053). The similarly adjusted results in subgroup analyses based on analyses of complete cases showed that this difference in systolic blood pressure was driven by male participants and by participants with a university education (see Supplementary material online, *Table S3*). LDL cholesterol levels differed marginally (0.1 mmol/L higher in the II-group) among those who were 60 years at baseline (*P* = 0.041), and at the same time LDL was 0.16 mmol lower in 50-year-old II-group participants (*P* = 0.033). No differences between the two groups were seen in subgroups by baseline risk category according to SCORE2.

**Table 2:** Differences between the II-group and the CI-group at the 6-year follow-up in complete-case analyses and in intention-to-treat analysis after imputation of missing data

	Complete-case results *n* = 2653			Intention-to-treat results *n* = 3532		
Outcomes	Difference	95% CI interval	*P*-values	Difference	95% CI interval	*P*-values
SCORE2	−0.17	[−0.35, 0.02]	0.08	−0.10	[−0.28, 0.09]	0.32
FRS	−0.41	[−0.98, 0.17]	0.17	−0.27	[−0.85, 0.31]	0.36
P-total cholesterol (mmol/L)	0.05	[−0.04, 0.13]	0.26	0.05	[−0.03, 0.13]	0.21
P-LDL cholesterol (mmol/L)	0.04	[−0.04, 0.12]	0.34	0.05	[−0.02, 0.13]	0.15
P-HDL cholesterol (mmol/L)	0.01	[−0.01, 0.03]	0.60	0.05	[−0.03, 0.13]	0.21
P-non-HDL cholesterol (mmol/L)	0.04	[−0.04, 0.13]	0.32	0.00	[−0.02, 0.03]	0.85
P-triglycerides^a^ (mmol/L)	1.00	[0.98, 1.03]	0.74	1.00	[0.98, 1.03]	0.77
Systolic blood pressure (mmHg)	−1.13	[−2.28, 0.02]	0.05	−1.33	[−2.56, −0.09]	0.04
Diastolic blood pressure (mmHg)	−0.11	[−0.80, 0.57]	0.74	−0.18	[−0.90, 0.53]	0.62
BMI (kg/m^2^)	−0.02	[−0.19, 0.15]	0.83	−0.12	[−0.37, 0.13]	0.34
Weight (kg)	0.02	[−0.48, 0.52]	0.94	−0.20	[−0.93, 0.54]	0.60
Waist circumference (cm)	−0.17	[−0.71, 0.38]	0.55	−0.34	[−1.02, 0.35]	0.33
	**RR**	**95% CI interval**	***P*-values**	**RR**	**95% CI interval**	***P*-values**
Smoking	0.94	0.77	1.14	1.11	[0.91, 1.35]	0.64
Diabetes	0.91	0.73	1.14	0.89	[1.09, 1.09]	0.45

Continuous variables were analysed with ANCOVA adjusted for baseline level, sex, and education. Negative results indicate lower level in the intervention group. Categorical variables were analysed using robust Poisson regression and presented as relative risk with 95% confidence intervals. The II-group received the VIPIVZA intervention at baseline and again at the 3-year follow-up. The CI-group underwent the first intervention at the 3-year follow-up

^a^Triglycerides are expressed as a ratio of the geometric mean instead of a difference.

SCORE2, the European Coronary Risk Evaluation 2; FRS, Framingham Risk Score; BMI body mass index.

**Figure 2:**
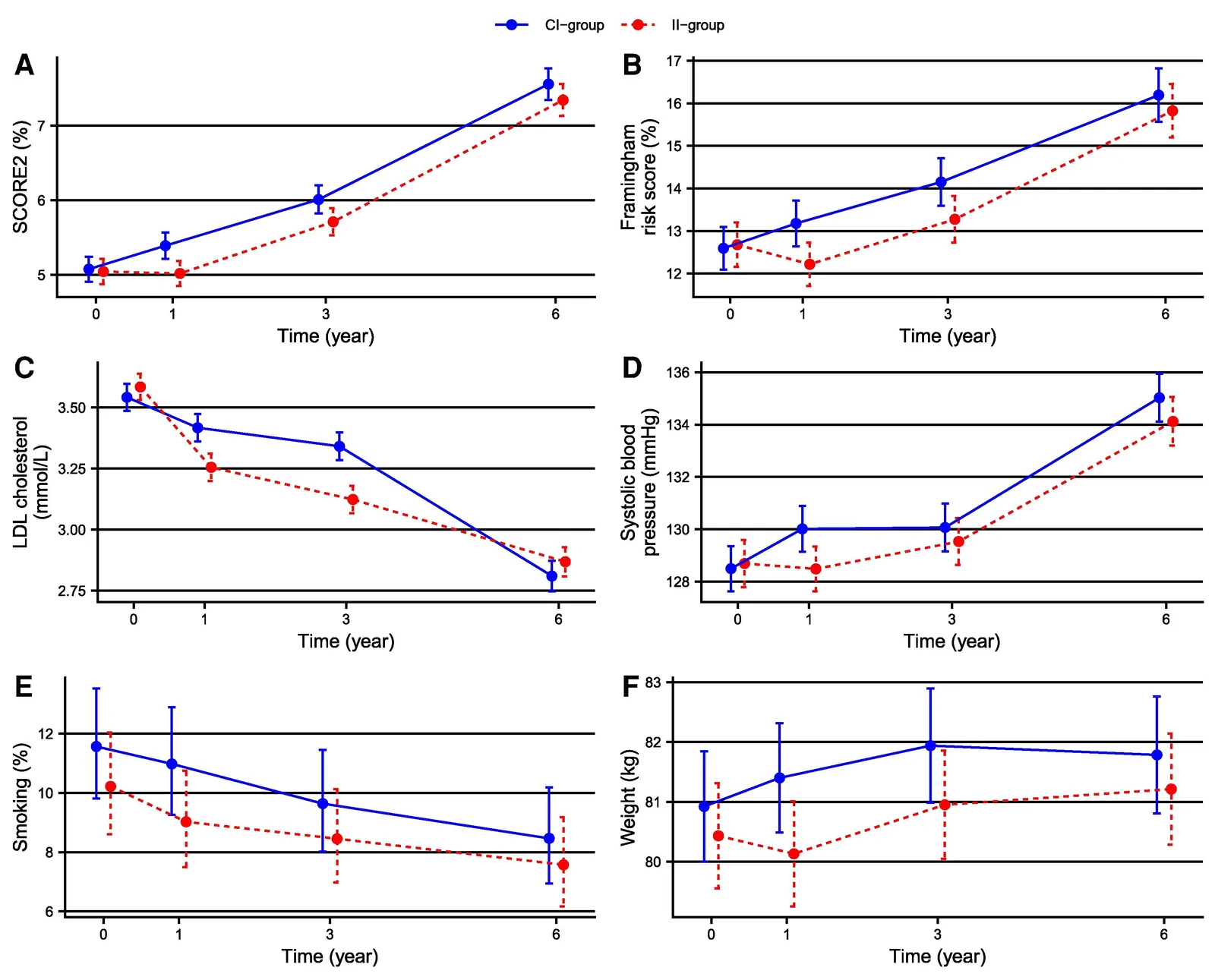
Crude time trends of SCORE2 (*A*), Framingham Risk Score (*B*), LDL cholesterol (*C*), systolic blood pressure (*D*), smoking (*E*), and waist circumference (*F*) in both groups from baseline over the 1-year and 3-year follow-up visits and up to the 6-year follow-up. SCORE2, the European Systematic Coronary Risk Evaluation 2.

*Figure 2* demonstrate the crude time trends from baseline to 6-year follow-ups of relevant variables in both groups among those 1254 II-group participants and 1194 CI-group participants who also provided data on risk factors and lifestyle at 1-and 3-year visits. After baseline, the lines for CVD risk scores and systolic blood pressure diverged initially, resulting in lower levels in the II-group after 1 year, but after the single-arm cross-over at the 3-year follow-up, the lines were increasing and virtually parallel with still slightly lower level in the II-group. By contrast, LDL levels also diverged after baseline in both groups, but with continued declining trends, that was most marked in the II-group after baseline and in the CI-group after the cross-over, and resulted in converging lines and similar LDL cholesterol levels after 6 years. The prevalence of smoking was initially overall around 10−12% in both groups and decreased over the 6-year period to around 8%. Weight increased slightly in both groups during the first 3 years and was thereafter rather stable.

**Figure 3:**
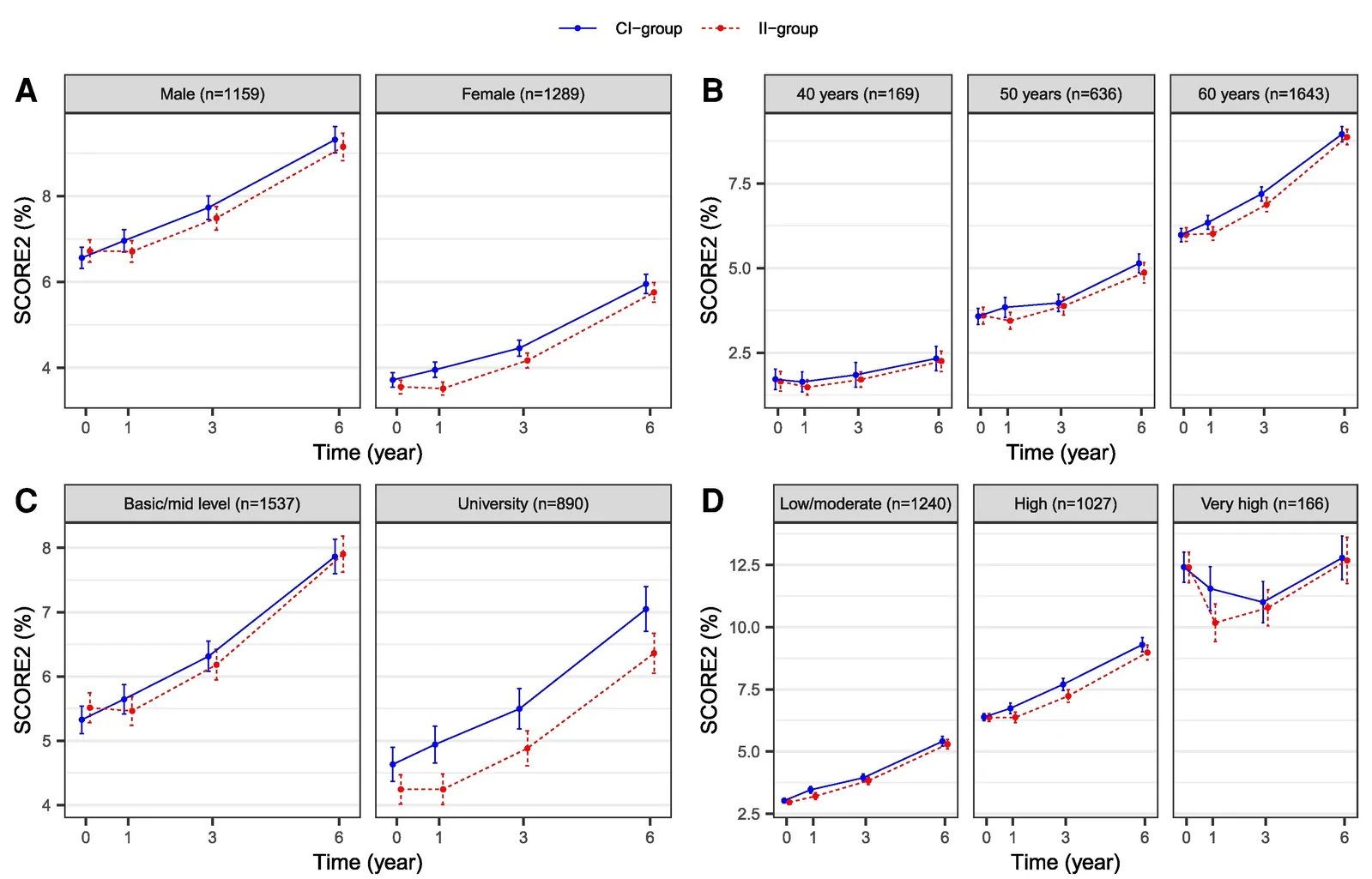
Crude time trends of SCORE2 in subgroups by sex (*A*), age groups (*B*), educational level (*C*), and risk of CVD according to SCORE2 at baseline (*D*) in both groups from baseline over the 1-year and 3-year follow-up visits and up to the 6-year follow-up. CVD, cardiovascular disease; SCORE2, the European Systematic Coronary Risk Evaluation 2.

**Figure 4:**
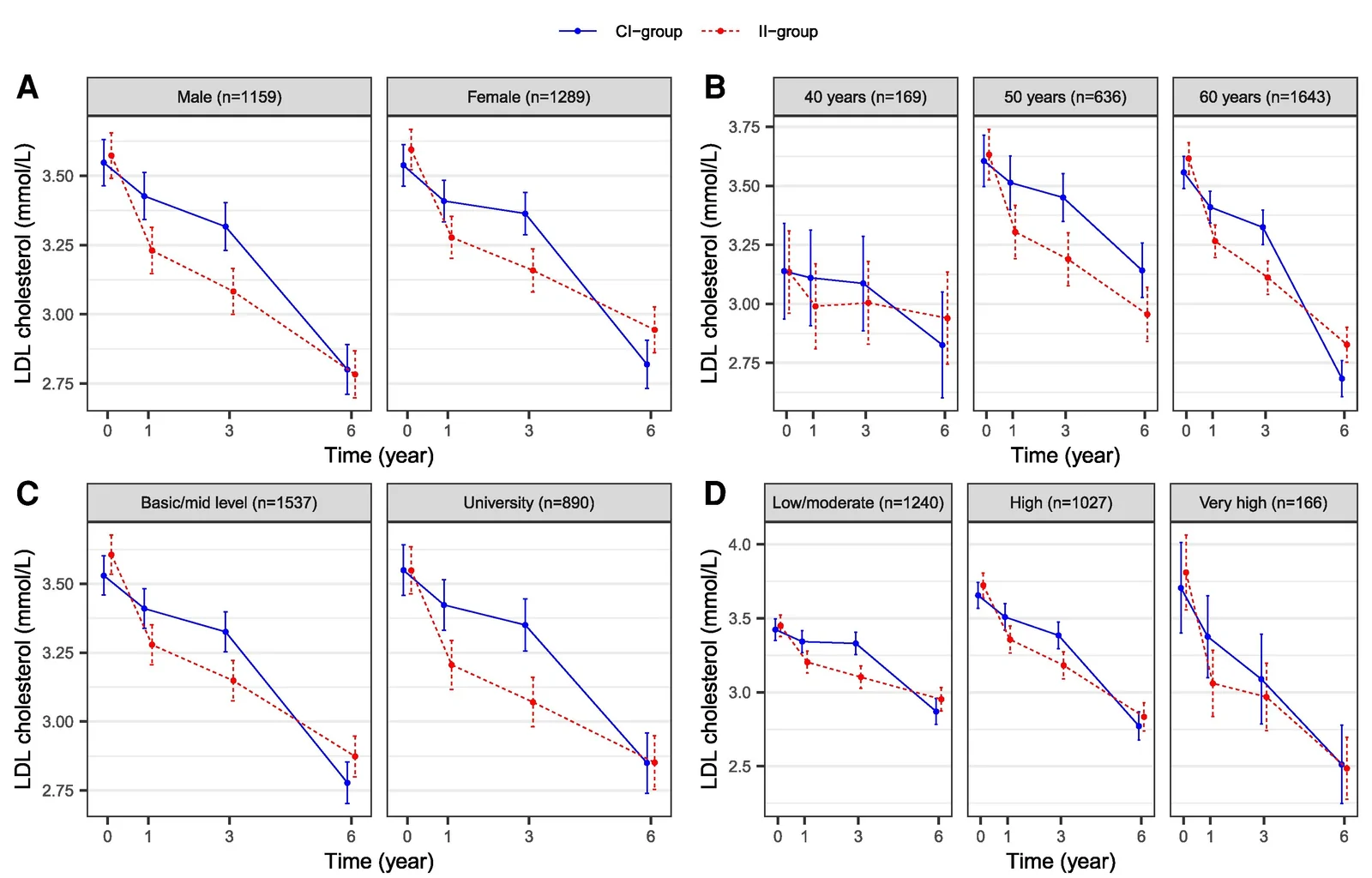
Crude time trends of LDL cholesterol levels in subgroups by sex (*A*), age group (*B*), educational level (*C*), and risk of CVD according to SCORE2 at baseline (*D*) in both groups from baseline over the 1-year and 3-year follow-up visits and up to the 6-year follow-up. CVD, cardiovascular disease; SCORE2, the European Systematic Coronary Risk Evaluation.

Time trends from baseline to the 6-year follow-up in subgroup analyses by sex (*A*), age at baseline (*B*), educational level (*C*), and baseline SCORE2 category (*D*) are shown for SCORE2 and LDL cholesterol in *Figures 3* and *4*, respectively, and for FRS, systolic blood pressure, smoking, and weight in Supplementary material online, *Figures S1*–*S4*, respectively. Overall, in all subgroups, the time trends were similar to those for the entire study population, and levels were higher among men, participants being 60 years old at baseline, lacking a university education, or at highest baseline risk category, according to SCORE2, compared to their counterparts. A few deviations from these patterns were noted. For example, in the highest base line risk category, time trends for SCORE2, FRS, and systolic blood pressure were U-shaped in both groups, resulting in similar levels (SCORE2) or even lower levels (FRS and systolic blood pressure) after 6 years compared to baseline levels. Also, no differences were seen between men and women and between educational groups in levels and trends of LDL, and, notably, the higher the CVD risk was at baseline, the higher the baseline LDL level and the steeper the decline, resulting in a reversed order between baseline risk categories in LDL cholesterol level after 6 years in both groups. Systolic blood pressure increased over time in both sexes. Among men, the trends diverged, resulting in constantly increasing gap between the II-group and the CI-group. By contrast, among females, systolic blood pressure levels increased, but there were no differences between the two groups at either baseline or after 6 years. Time trends for smoking differed largely by baseline CVD risk category. Thus, among those at very high risk at baseline, the initial prevalence of ∼50% declined to ∼30% over the 6 years, while the prevalence of ∼3% was stable in the lowest risk category. Weight patterns were similar in most subgroups with only small changes observed during 6 years. Of note was considerable weight gain in the 40-year-old participants, a more modest increase among those aged 50 years at baseline, and weight maintenance among the 60-year-olds.

## Discussion

Our main result at 6 years, after the single-arm cross-over, is that no statistically significant difference in CVD risk was observed between the groups. The outcome trajectories suggest that the CI-group experienced effects aligning to those previously reported in the II-group. Even though the general pattern is that the CI-group still had lower point estimates in risk factors than the II-group, the statistically significant differences in CVD risk, previously shown after 1 and 3 years,^7,8^ were no longer seen after 6 years. Since the intervention effect was similar in both groups, our hypothesis was accepted, and previous reports were retrospectively supported. With reduced sample size in the complete-case analysis results at 6 years compared to 1 and 3 years due to the attrition rate, lower statistical power is expected, but the imputed analyses similarly found few statistically significant differences.

Secondly, the trend analyses demonstrate a strong intervention effect on lipid levels. This supports previously presented trends over 5 years regarding prescriptions.^10^ Also the repeated intervention in the II-group was followed by additionally increased initiation of lipid-lowering medication, and in parallel an almost complete catch-up in the CI-group was demonstrated after their first intervention at the 3-year follow-up.^20^ The decrease in LDL cholesterol in the CI-group was more rapid compared to the corresponding decrease in the II-group after their first intervention at baseline. This illustrates that at the time for the 3-year follow-up, new routines for managing the CUS results were established in primary care, and consequently, the prescribing GPs were more active than during the implementation in the 3-year inclusion period. Gradually increased preventive actions were previously also observed.^21^ This advantage for the CI-group may have balanced the fact that, contrary to in the II-group, the personalized ultrasound report was not sent out again 6 months after the 3-year follow-up and no reminders were sent. Moreover, a slight additional increase in initiation of statins was also seen in the II-group after the 3-year follow-up, demonstrating the impact of repeated intervention,^20^ as well as improved adherence to statin treatment among those who already used it before baseline.^22^ We therefore believe that a large part of impact of the VIPVIZA intervention on the risk of CVD was attributable to markedly increased use of statins. Contributing factors should be that both participants and primary care physicians were targeted. In this pragmatic trial, preventive pharmacological treatments were in the hands of primary care without involvement of the research team. This could be contrasted with other imaging trials, where statins were prescribed by the research team to all intervention group participants,^23^ or to intervention group participants with demonstrated subclinical atherosclerosis,^24^ or GPs were recommended to prescribe statins in case of abnormal imaging results.^25^

Interestingly, in the small category at highest baseline risk of CVD, the time trends were U-shaped for risk scores and systolic blood pressure, resulting in similar levels after 6 years as at baseline, despite increasing age.

The fact that men seem to benefit more than women regarding blood pressure is supported by a review showing higher percentage of nonadherence to antihypertensive medication in women than in men.^26^ However, other factors need further exploration, for example differences between sexes when it comes to lifestyle modification.

Current results and previous results^7,8^ are supported by studies focused on modifiable psychological factors, which could in turn impact on preventive measures. Thus, the combination of a strong cognitive response (improved risk perception and efficacy beliefs) and strong emotional reactions, irrespective of positive (positive surprise) or negative (worry, anxiety) valence, was associated with the largest improvement in lifestyle habits.^18^ Also, II-group participants perceived the risk of CVD to be higher after 3 years compared to those who had not yet undergone the intervention. The severity of the colour-coded and age-related message about atherosclerosis was also associated with participants’ CVD risk perception.^17^ However, results concerning effect on efficacy beliefs (participants’ perception of their own ability to fulfil recommended preventive measures and if they believe that these measures may reduce their risk) were not conclusive.^17,18,27^ Thus, VIPVIZA has contributed to answering how and why the intervention, including visualization of imaging results, may impact CVD risk.

Comparing trials using imaging of subclinical atherosclerosis to improve CVD prevention is hampered due to several reasons. A summary regarding some recently reported trials is shown in Supplementary material online, *Table S4*. First, *imaging techniques*, *communication strategies, and format* used to inform participants about the imaging results differ, as does *whether and how imaging results are available in primary care.* Secondly and importantly, imaging trials use very different *additional intervention components*. Treatment with statins and antihypertensive drugs is usually in the hands of primary care, with or without information about imaging results (see above). In other trials, statins were prescribed free of cost by the study team to all,^23^ or to some intervention group participants,^24^ or GPs were recommended to prescribe statins.^25^  *Personalized lifestyle intervention* provided by the research team as part of the intervention can be given, but vary largely by intensity and frequency, or intervention group participants may be advised to contact their GPs, who are asked to follow CVD prevention guidelines. Additional technical equipment, such as physical activity trackers and stand–sit workstations, can also be used. *Control groups are managed differently*, for example if imaging is performed, and if and how control participants and their GPs get imaging results. Referral to or prompting control participants to contact their GP in case of abnormal risk factor results is practiced in most trials. Finally, trials also differ largely in *case-mix*, for example regarding source population, age, and sex, and representing groups of the population at different levels of CVD risk.

Besides comparisons of intervention effects, these obvious disparities between imaging trials and often lack of, or incomplete, information about components used in complex intervention trials hampers also the possibility to clarify the intervention mechanisms and to translate research evidence into practice.^28,29^ Indeed, a recent meta-review evaluating behavioural change techniques within CVD prevention trials found that none of the 15 included reviews reported mechanism of actions.^30^ Standardized and structured reporting explicitly defining and assessing active intervention components related to modifiable mediating psychological factors would enable comparisons between complex imaging trials and allow conclusions to be drawn about the effect of specified intervention components, among which imaging, including the format for information about imaging results, is not the only intervention component.^6,31,32^

### Strengths and limitations

A strength is its pragmatic design, which increases clinical relevance and external validity. The participation rate—76%—should be considered high, taking into account that the 6-year follow-up was performed during the COrona VIrus Disease of 2019 (COVID-19) pandemic, including accompanying restrictions to daily life. Contributing to the high rate is the fact that the same methodology was applied, and the same team of research nurses and ultrasonographers administered and conducted the examinations over the entire county and course of the study. However, due to the pandemic’s restrictions, the time between 3-year and 6-year follow-up visits was considerably delayed for many participants even up to 7–8 years.

A limitation is that smoking was self-reported. Also, we did not have information on comorbidities and treatments for comorbidities that could have impact CVD risk, participation, and preventive measures during the study period, for example cancers. A limitation is that after the delayed and repeated intervention, VIPVIZA cannot directly estimate the intervention effect, since there is no comparator.

### Conclusions

The effect of the first intervention, which was provided at baseline in the II-group and after 3 years in the CI-group, was similar in the two groups. After 6 years, there was no statistically significant difference in 10-year risk of CVD between the two groups. We observed a strong effect on cholesterol levels in both groups.

## Supplementary Material

oeag047_Supplementary_Data

## Data Availability

The data and statistical code underlying this article will be shared upon reasonable request to the PI for VIPVIZA, e-mail: patrik.wennberg@umu.se.

## Lead author biography

Margareta Norberg, MD, PhD, is specialized in family medicine; involved in primary CVD prevention at a public health level during 2007–2018 in the Västerbotten Intervention Programme, which aims at CVD prevention and is integrated in primary healthcare in Västerbotten County, Northern Sweden; and is active in the steering group of the VIPVIZA trial since it commenced in 2012.
