# Supplementary material for: Evaluation after delayed and repeated intervention in the VIPVIZA-extended randomized controlled trial: beneficial results 6 years after baseline
Source: Eur Heart J Open. 2026 Apr 13;6(2):oeag047. doi: 10.1093/ehjopen/oeag047 (PMC13075482; doi:10.1093/ehjopen/oeag047)
Supplement: oeag047_Supplementary_Data [file oeag047_supplementary_data.zip › Updated March 27 2026 Clean Revised material VIPVIZA Tables and Legends to Supplementary Figures.pdf]

SUPPLEMENTARY TABLES VIPVIZA

Supplementary Table1. Baseline characteristics in the dropout analyses of six-year follow-up. Dropouts at six-year follow-up comprised those who had withdrawn baseline informed consent, did not consent to the six-year follow-up, did not show up, were excluded due to participation in other study, had migrated out of the county, or had died.

	**II Group (n=1749)**			**CI Group (n=1783)**		
	**Those who dropped out (n=410)**	**Those who participated in the 6-year FU (n=1339)**	**p-value**	**Those who dropped out (n=460)**	**Those who participated in the 6-year FU (n= 1323)**	**p-value**
SCORE2 Risk estimates (10-year risk in %)[1]	5.34 (3.5)	5.0 (3.1)	0.051	5.36 (3.49)	5.08 (2.97)	0.102
Framingham Risk score (10-year risk in %)[1]	13.7 (10.3)	12.7 (9.4)	0.068	13.61 (9.84)	12.62 (8.85)	**0.046**
Sex, women n (%)	233 (56.8)	707(52.8)	0.157	232 (50.4)	698 (52.8)	0.416
Age group n (%)			**0.038**			**<0.001**
40 years	31 (7.6)	103(7.7)		56 (12.2)	86 (6.5)	
50 years	134 (32.7)	352(26.3)		140 (30.4)	352 (26.6)	
60 years	245 (59.8)	884(66.0)		264 (57.4)	885 (66.9)	
Basic/mid-level education[2] n (%)	296 (72.7)	847(63.8)	**<0.001**	310 (68.9)	827 (63.0)	**<0.001**
Married/cohabiting n (%)	297 (72.8)	1075(81.0)	**<0.001**	309 (68.5)	1056 (80.7)	**<0.001**
Waist circumference cm[1]	98.79 (15.0)	95.38 (12.4)	**<0.001**	99.2 (13.5)	96.1 (13.5)	**<0.001**
Weight kg[1]	84.2 (20.6)	80.6 (16.0)	**<0.001**	85.0 (17.6)	81.2 (16.5)	**<0.001**
Fasting P-glucose mmol/L	5.5 (1.5)	5.3 (1.1)	**<0.001**	5.5 (1.5)	5.4 (1.2)	**0.015**
Diabetes n(%)	33 (8.1)	66 (4.9)	**0.036**	32 (7.0)	68 (5.2)	0.190
Systolic blood pressure mmHg[1]	131.9 (17.5)	128.81 (16.3)	**<0.001**	131.3 (17.5)	128.5 (15.3)	**<0.001**
Diastolic blood pressure mmHg[1]	84.6 (11.4)	82.11 (10.5)	**<0.001**	83.8 (11.3)	82.26 (9.9)	**0.003**
Hypertension n(%)	245(59.8)	673 (50.3)	**<0.001**	269 (58.5)	641 (48.5)	**<0.001**
P-Total-Cholesterol mmol/L[1]	5.58 (1.1)	5.61 (1.1)	0.579	5.6 (1.1)	5.6 (1.1)	0.390
P-HDL-Cholesterol mmol/L[1]	1.37 (0.4)	1.40 (0.4)	0.253	1.4 (0.4)	1.4 (0.4)	0.104
P-LDL-Cholesterol mmol/L[1]	3.5 (1,0)	3.6 (1.0)	0.374	3.5 (1.0)	3.55 (1.0)	0.378
P-non-HDL-Cholesterol[1]	4.2 (1.1)	4.2 (1.1)	0.911	4.23 (1.1)	4.2 (1.1)	0.221
Triglycerides mmol/L[1]	1.6 (0.9)	1.5 (1.0)	**0.044**	1.6 (1.0)	1.5 (0.9)	**0.010**
Smoking daily/occasionally n(%)	70 (17.1)	137 (10.3)	**0.002**	84 (18.4)	155 (11.7)	**<0.001**

[1] Mean (SD)

[2] Self-reported basic or midlevel education. All other have academic/university level.

[3] Self-reported known diabetes or f-P-glucose ≥7.0 mmol/L

[4] Self-reported use of antihypertensive medication or systolic blood pressure ≥140 mmHg or diastolic blood pressure ≥90 mmHg

Abbreviations: SCORE2 the European Coronary Risk Evaluation 2, HDL High Density Lipoprotein, LDL Low Density Lipoprotein.

Supplementary Table 2. Missing data among participants at the six-year follow-up.

Characteristics	II-group	CI-group
	6-year Follow-up (n=1339) n (%)	6-year Follow-up (n=1323) n (%)
SCORE2 Risk estimates	26 (1.9)	34 (2.6)
Framingham Risk score	26 (1.9	34 (2.6)
P-Total Cholesterol	15 (1.1	13 (1.0)
P-HDL Cholesterol	15 (1.1)	13 (1.0)
P-LDL Cholesterol	32 (2.4)	29 (2.2)
P-non-HDL Cholesterol	15 (1.1)	13 (1.0)
P-Triglycerides	15 (1.1)	13 (1.0)
Systolic blood pressure	14 (1.0)	17 (1.3)
Diastolic blood pressure	14 (1.0)	17 (1.3)
Fasting P-glucose	23 (1.7)	30 (2.3)
Diabetes	23 (1.7)	30 (2.3)
Body weight	14 (1.0)	19 (1.4)
Smoking daily/occasionally	5 (0.4)	5 (0.4)
Education level (baseline data)	11 (0.8)	11 (0.8)

Abbreviations: SCORE2 the European Coronary Risk Evaluation 2, HDL High Density Lipoprotein, LDL Low Density Lipoprotein.

Supplementary Table 3. Subgroup analyses showing differences between the II-group and the CI-group at the six-year follow-up in complete case analyses. Continuous variables were analyzed with ANCOVA adjusted for baseline level. Negative results indicate lower level in the CI group. Categorical variables were analysed using robust Poisson regression and presented as relative risk with 95% confidence intervals.

**Subgroups by sex**									
	**Male n=1251**			**Female n=1402**					
**Characteristic**	**Difference**	**95% CI interval**	**p-vales**	**Difference**	**95% CI interval**	**p-vales**			
SCORE2	-0.31	[-0.63, 0.01]	0.06	-0.01	[-0.22, 0.20]	0.90			
FRS	-0.97	[-2.00, 0.07]	0.07	0.09	[-0.47, 0.65]	0.75			
P-Total cholesterol (mmol/L)	0.01	[-0.11, 0.13]	0.88	0.08	[-0.03, 0.2]	0.17			
P-LDL-cholesterol (mmol/L)	-0.01	[-0.12, 0.10]	0.81	0.08	[-0.03, 0.19]	0.13			
P-HDL-cholesterol (mmol/L)	0.02	[-0.01, 0.05]	0.22	-0.01	[-0.04, 0.02]	0.71			
P-Non-HDL-cholesterol (mmol/L)	-0.01	[-0.14, 0.11]	0.82	0.09	[-0.02, 0.21]	0.12			
P-Triglycerides[3] (mmol/L)	0.99	[0.95, 1.03]	0.71	1.02	[0.98, 1.05]	0.37			
Systolic blood pressure (mmHg)	-1.68	[-3.30, -0.06]	0.04	-0.64	[-2.27, 1.00]	0.44			
Diastolic blood pressure (mmHg)	-0.66	[-1.68, 0.37]	0.21	0.40	[-0.51, 1.31]	0.39			
BMI (kg/m^2)	-0.06	[-0.28, 0.17]	0.62	0.01	[-0.24, 0.27]	0.93			
Weight (kg)	-0.17	[-0.88, 0.55]	0.65	0.18	[-0.51, 0.87]	0.61			
Waist (cm)	-0.09	[-0.84, 0.66]	0.82	-0.22	[-1.00, 0.56]	0.57			
Smoking	0.91	[0.60, 1.37]	0.64	0.97	[0.67, 1.39]	0.88			
Diabetes	0.90	[0.65, 1.24]	0.52	0.92	[0.65, 1.24]	0.67			
**Subgroups by education**									
	**Basic/Middle education n=1667**			**University/Academic education n=964**					
**Characteristic**	**Difference**	**95% CI interval**	**p-vales**	**Difference**	**95% CI interval**	**p-vales**			
SCORE2	-0.09	[-0.34, 0.16]	0.46	-0.26	[-0.54, 0.02]	0.07			
FRS	-0.23	[-1.02, 0.55]	0.56	-0.60	[-1.39, 0.19]	0.14			
P-Total cholesterol (mmol/L)	0.06	[-0.04, 0.17]	0.24	0.03	[-0.11, 0.17]	0.69			
P-LDL-cholesterol (mmol/L)	0.07	[-0.03, 0.17]	0.16	-0.01	[-0.14, 0.12]	0.87			
P-HDL-cholesterol (mmol/L)	0.00	[-0.03, 0.02]	0.95	0.02	[-0.02, 0.05]	0.38			
P-Non-HDL-cholesterol (mmol/L)	0.07	[-0.04, 0.17]	0.22	0.01	[-0.13, 0.15]	0.89			
P-Triglycerides[3] (mmol/L)	1.00	[0.97, 1.03]	0.98	1.01	[0.97, 1.06]	0.50			
Systolic blood pressure (mmHg)	-0.39	[-1.86, 1.07]	0.60	-2.42	[-4.28, -0.56]	0.01			
Diastolic blood pressure (mmHg)	-0.12	[-0.99, 0.75]	0.79	-0.09	[-1.19, 1.01]	0.87			
BMI (kg/m^2)	0.07	[-0.15, 0.30]	0.52	-0.18	[-0.43, 0.07]	0.15			
Weight (kg)	0.25	[-0.41, 0.92]	0.46	-0.38	[-1.11, 0.34]	0.30			
Waist (cm)	0.07	[-0.64, 0.78]	0.84	-0.58	[-1.41, 0.25]	0.17			
Smoking	1.00	[0.73, 1.38]	0.98	0.78	[0.43, 1.38]	0.40			
Diabetes	0.87	[0.66, 1.16]	0.35	1.02	[0.64, 1.64]	0.93			

**Subgroups by age group at baseline**									
	**40 years old n=187**			**50 years old n=704**			**60 years old n=1764**		
**Characteristic**	Difference	95% CI interval	p-value	Difference	95% CI interval	p-value	Difference	95% CI interval	p-vales
SCORE2	-0.11	[-0.42, 0.20]	0.48	-0.04	[-0.33, 0.26]	0.79	-0.15	[-0.37, 0.06]	0.17
FRS	-0.48	[-1.22, 0.26]	0.21	-0.35	[-1.30, 0.60]	0.47	-0.31	[-1.06, 0.44]	0.42
P-Total cholesterol (mmol/L)	0.12	[-0.16, 0.40]	0.41	-0.11	[-0.27, 0.05]	0.17	0.10	[0.00, 0.21]	0.06
P-LDL-cholesterol (mmol/L)	0.17	[-0.08, 0.42]	0.19	-0.16	[-0.31, -0.01]	0.03	0.10	[0.00, 0.20]	0.04
P-HDL-cholesterol (mmol/L)	0.05	[-0.03, 0.12]	0.21	0.03	[-0.01, 0.07]	0.16	-0.01	[-0.03, 0.02]	0.46
P-Non-HDL-cholesterol (mmol/L)	0.06	[-0.22, 0.34]	0.66	-0.14	[-0.3, 0.02]	0.09	0.11	[0.01, 0.22]	0.04
P-Triglycerides[3] (mmol/L)	0.92	[0.82, 1.03]	0.15	1.00	[0.94, 1.05]	0.88	1.02	[0.98, 1.05]	0.31
Systolic blood pressure (mmHg)	-1.57	[-4.91, 1.76]	0.35	-0.39	[-2.58, 1.80]	0.73	-1.18	[-2.62, 0.27]	0.11
Diastolic blood pressure (mmHg)	-0.19	[-2.56, 2.17]	0.87	0.08	[-1.28, 1.45]	0.90	-0.08	[-0.91, 0.75]	0.85
BMI (kg/m^2)	-0.66	[-1.33, 0.01]	0.05	0.02	[-0.36, 0.40]	0.92	0.02	[-0.18, 0.21]	0.87
Weight (kg)	-1.69	[-3.8, 0.42]	0.12	0.06	[-1.04, 1.17]	0.91	0.13	[-0.42, 0.68]	0.64
Waist (cm)	-1.16	[-3.24, 0.92]	0.27	0.16	[-0.99, 1.31]	0.78	-0.16	[-0.80, 0.48]	0.63
Smoking (%)	1.76	[0.597, 5.64]	0.32	1.13	[0.67, 1.91]	0.65	0.79	[0.56, 1.12]	0.19
Diabetes (%)	0.50	[0.06, 3.08]	0.45	1.23	[0.74, 2.06]	0.43	0.85	[0.64, 1.12]	0.26
**Risk level at baseline according to SCORE2**									
	**Low/Moderate risk n=1342**			**High risk n=1109**			**Very high risk n=184**		
**Characteristic**	Difference	95% CI interval	p-value	Difference	95% CI interval	p-value	Difference	95% CI interval	p-value
SCORE2	-0.02	[-0.18, 0.14]	0.80	-0.23	[-0.55, 0.09]	0.16	-0.22	[-1.38, 0.95]	0.71
FRS	0.03	[-0.44, 0.51]	0.89	-0.80	[-1.85, 0.24]	0.13	-0.28	[-4.06, 3.49]	0.88
P-Total cholesterol (mmol/L)	0.09	[-0.03, 0.20]	0.14	0.03	[-0.10, 0.16]	0.66	-0.13	[-0.45, 0.19]	0.43
P-LDL-cholesterol (mmol/L)	0.06	[-0.05, 0.16]	0.28	0.02	[-0.10, 0.15]	0.71	-0.04	[-0.34, 0.26]	0.81
P-HDL-cholesterol (mmol/L)	0.01	[-0.02, 0.05]	0.36	0.00	[-0.03, 0.03]	0.82	-0.02	[-0.08, 0.05]	0.65
P-Non-HDL-cholesterol (mmol/L)	0.07	[-0.04, 0.19]	0.21	0.03	[-0.10, 0.16]	0.66	-0.09	[-0.42, 0.24]	0.59
P-Triglycerides[3] (mmol/L)	1.02	[0.98, 1.06]	0.27	1.00	[0.96, 1.04]	0.91	0.93	[0.83, 1.05]	0.25
Systolic blood pressure (mmHg)	-1.04	[-2.58, 0.51]	0.19	-1.12	[-2.92, 0.69]	0.22	-1.10	[-6.49, 4.30]	0.69
Diastolic blood pressure (mmHg)	0.11	[-0.80, 1.01]	0.81	-0.04	[-1.13, 1.06]	0.95	-0.85	[-3.93, 2.23]	0.59
BMI (kg/m^2)	-0.13	[-0.37, 0.11]	0.27	0.17	[-0.09, 0.43]	0.19	-0.18	[-0.83, 0.48]	0.60
Weight (kg)	-0.34	[-1.02, 0.33]	0.31	0.56	[-0.22, 1.34]	0.16	-0.35	[-2.50, 1.80]	0.75
Waist (cm)	-0.65	[-1.4, 0.11]	0.09	0.43	[-0.49, 1.26]	0.31	-0.38	[-2.70, 1.94]	0.75
Smoking (%)	0.78	[0.43, 1.38]	0.40	0.84	[0.58, 1.21]	0.36	1.03	[0.58, 1.79]	0.93
Diabetes (%)	1.02	[0.64, 1.64]	0.94	0.89	[0.63, 1.24]	0.48	0.87	[0.54, 1.41]	0.58

Abbreviations: SCORE2 the European Coronary Risk Evaluation 2, FRS Framingham Risk Score, LDL Low Density Lipoprotein, HDL High Density Lipoprotein, BMI Body Mass Index.

Supplementary Table 4. A few recent imaging trials for primary CVD prevention: Study population, imaging technique, modality for information about imaging results, additional intervention components and results.

	**VIPVIZA** [1-7]	**CAUGHT-CAD** [8-10]	**TANSNIP-PESA** [11,12]	**DANCAVAS** [13,14]	**ROBINSCA** [15-19]
Population	Sweden. Population based. Low/intermediate risk of CVD. 40, 50 , 60 years old with at least one traditional CVD risk factor. RCT.	Australia. From community work places and response to information in media. Asymptomatic statin-free , 40-70 years, calculated intermediate CVD risk and family history of premature coronary artery disease, CAC score 1-400 (medium risk). RCT.	Spain. Worksite: Employees at Banco of Santander, Madrid Having subclinical atherosclerotic plaque burden, 40-60 years. RCT.	Denmark. Population based. Men 65-74. RCT.	Netherlands. Population based. Men 45-74 years and women 55-74 years. Asymptomatic, at expected high risk of CVD: Waist ≥ 102 cm (men)/≥88 cm (women), BMI≥30, smoking, f amily history of CHD. Arms, screening : A: Traditional risk factors B: CAC C: Control. RCT, compares Arm A vs arm B.
Imaging technique in the intervention group	CUS	CAC	CAC and CUS and ultrasound examination of iliofemoral arteries and infrarenal aorta,	CAC and Truncus CT to identify aortic/iliac aneurysm, including screening for atrial fibrillation	Arm B: CAC[2] and calculation of the Agatston score
Communication strategies to intervention group participants about imaging results	Pictorial color-coded and age-related information about CIMT (Vascular age) and plaque+ the same repeated once	Raw images and the CAC score discussed with a nurse 4 times during first three months, after that on request at	Written information and verbal feed back about CAC score, number of plaques and territories affected on ultrasound, no	If CAC score >median , or aortic/iliac aneurysm verbal information including clinical implications. If not: written information via ordinary mail.	Arm B: Participants were informed about their risk status and -If Agatston score was low: (<100) No action recommended.

		biannual visits during 36 months.	specific mention on low or high burden		- If high (100-399) or very high (≥400): Referral to GP
	**VIPVIZA** [1-5]	**CAUGHT-CAD** [8,9]	**TANSNIP-PESA** [12]	**DANCAVAS** [13,14]	**ROBINSCA** [16]
Pictorial information	yes	Yes	No	No	No
Information to usual care about imaging results	The same as to the intervention participants	GPs informed about the CAC_score, predicted CVD risk, initiation of statins by the study cardiologist and, if indicated, antihypertensives	No	GPs informed electronically about all screening results	Arm B: GPs informed about the CAC-score if high or very high CAC score(≥100)
Imaging technique in the control group	Same as the intervention group	Same as in the intervention group	Same as the intervention group	No imaging	No imaging<br>Arm A: Risk score<br>Arm C: No screening
Information to control participants about imaging results	None	None	Same written information as to the intervention group but no verbal feed back	n.a.	n.a.<br>Arm A: Participants informed about their risk SCORE and if medium or high risk advised to consult their GP
Information to usual care about imaging results in control participants	None	Information that participants' had an intermediate CVD risk, that they had identified subclinical atherosclerosis (but not the CAC_score) and participation in a 36-month study	No	n.a.	n.a.<br>Arm A: GPs informed about the risk status and asked to follow guidelines<br>if high risk (≥20) or medium risk (10-19) + ≥1 risk factor: lifestyle modification and pharmacological treatment if systolic

					blood-pressure ≥140 and/or LDL≥2.5 mmol/l.
	**VIPVIZA** [1-5]	**CAUGHT-CAD** [8,9]	**TANSNIP-PESA** [12]	**DANCAVAS** [13,14]	**ROBINSCA** [16]
Additional intervention components provided to the intervention group	1/Risk factor screening and individual MI in dialogue with a clinical nurse in VIP[3] before invitation to the trial (same as in the control group).<br>2/Information to each GP receiving an ultrasound report that presence of plaque indicates very high risk.<br>3/One-year follow-up; Risk factor measurements and questionnaires on lifestyle, results and general recommendations following guidelines given to participants and their GP.<br>4/Pharmacological treatment managed by GPs according to	1/Statins free of cost prescribed by the study cardiologist to all intervention participants, and if untreated hypertension antihypertensives prescribed.<br>2/Nurse-led provision of personalized dietary, weight, smoking, and physical activity goals with support to self-management at baseline, 2w, 4w, 3m and thereafter biannual during 36 months.<br>3/Improved coordination of subjects' care providers.	1/Work-site intensive personalized lifestyle intervention based om MI delivered by psychologists trained in MI during 12 sessions<br>2/Wrist worn physical activity tracker with smartphone connectivity<br>3/ A sit-stand work-station<br>4/ pharmacological treatment within standard care	1/If positive findings (CAC > median, aneurysm, PAD or atrial fibrillation: recommendation of lifestyle modification + prescription of ASA and statin.<br>2/Follow-up of aneurysm, echocardiography if atrial fibrillation.<br>3/Referral to GP if: Systolic blood pressure ≥160, HbA1c ≥48, Cholesterol ≥8.	Arm B: If high or very high CAC score(≥100) GPs recommended to start pharmacological treatment: ACE-inhibitors and statins irrespective of blood pressure and cholesterol levels

	guidelines, no specific recommendation.				
	**VIPVIZA** [1-5]	**CAUGHT-CAD** [8,9]	**TANSNIP-PESA** [12]	**DANCAVAS** [13,14]	**ROBINSCA** [16]
Additional intervention components provided to the control group	1/Risk factor screening and individual MI in dialogue with a clinical nurse in VIP before invitation to the trial (Same as in the intervention group).<br>2/One-year follow-up: Risk factor measurements and questionnaires on lifestyle, results and general recommendations following guidelines sent to participants and their GP.<br>3/ Standard care managed by primary care	Standardized CV risk information and education on modifiable risk factors at biannual visits during 36 months. Pharmacological treatment of diabetes, hypertension and dyslipidemia managed by subjects' GP according to guidelines	Standard care	n.a. (No screening)	Arm A: Participants informed about their risk status and if low risk score no action, if medium or high risk score participants advised to consult GP<br><br>Arm C: No screening

Reported results	Significant difference between groups in SCORE and FRS after 1 and at 3 years to the benefit of the control group, and reduced progression of CIMT in the control group at 3 years. Increased initiation of statins among statin-naive and improved adherence to statins among those who used statins at baseline was seen in the intervention group.<br>Risk perception was improved in the intervention group.<br>An interaction between high cognitive and high emotional response to the intervention was associated with more lifestyle change over 3 years.	After 1 year the risk had decreased in the intervention group and increased in the control group. LDL decreased in the intervention group, was unchanged in the control group.<br>At 3 years: The reduction in FRS and LDL was greater in the control group.<br>Also within the intervention group, sustained recollection of the CAC image was associated to greater reductions in systolic blood pressure and waist circumference.	Significant improvement in CV health and behavioral metrics at 1 year, but not at 3 years. Over 3 years effect was seen only among participant having low baseline SA	After 5.6 years no statistically significant effect on total mortality HR 0.95 (0.90, 1.00), but among men aged 64-69 years HR 0.89 (0.83, 0,96).	Report 2019: Participants in Arm B (CAC) contacted their GP more often and had higher adherence to pharmacological preventive treatment compared to those in Arm A (risk factor score).<br>Report 2020: CAC scoring compared to SCORE classified significantly fewer at increased risk, and less preventive treatment was indicated.<br>Report 2024: CAC compared to risk assessment has no short-term detrimental effect on Health-related Quality of Life and anxiety (within 5-7 days after screening).<br>Receiving a result after 2-3 weeks indicating increased risk according to CAC was associated to increased anxiety.

Abbreviations: VIPVIZA Västerbotten Intervention Programme VisualiZation of asymptomatic Atherosclerotic disease for optimum cardiovascular prevention, CAUGHT-CAD The Coronary Artery calcium score: Use to Guide management of HerediTary Coronary Artery Disease, TANSNIP-PESA Trans-Atlantic Network to study Stepwise Non-invasive Imaging as a tool for CVD Prognosis and prevention- The Progression and Early detection of Subclinical Atherosclerosis, DANCAVAS The DANish CardioVAScular Screening Trial , ROBINSCA Risk Or Benefit IN Screening for CArdiovascular Diseases

CUS Carotid artery ultrasound examination, CT Computed Tomography, CAC Computed Tomography for evaluation of coronary artery calcium, VIP Västerbotten Intervention Programme [20], PAD Peripheral arterial disease, SCORE European Systematic Coronary Risk Evaluation[21], SCORE2 European Systematic Coronary Risk Evaluation 2, FRS Framingham risk score, CIMT Carotid Intima Media Thickness, GP General Practitioner, LDL Low Density Lipoprotein cholesterol , ASA Acetylsalicylic Acid, ACE-inhibitor Angiotensin-converting-enzyme inhibitor, HR Hazard ratio, SA Subclinical atherosclerotic plaque burden, HbA1c Haemoglobin A1c, glycated haemoglobin A1c.

References

1. Naslund U, Ng N, Lundgren A, Fharm E, Gronlund C, Johansson H, et al. Visualization of asymptomatic atherosclerotic disease for optimum cardiovascular prevention (VIPVIZA): a pragmatic, open-label, randomised controlled trial. *Lancet* 2019;**393**:133-142. doi: https://doi.org/10.1016/S0140-6736(18)32818-6
2. Bengtsson A, Norberg M, Ng N, Carlberg B, Gronlund C, Hultdin J, et al. The beneficial effect over 3 years by pictorial information to patients and their physician about subclinical atherosclerosis and cardiovascular risk: Results from the VIPVIZA randomized clinical trial. *Am J Prev Cardiol* 2021;**7**:100199. doi: https://doi.org/10.1016/j.ajpc.2021.100199
3. Andersson EM, Johansson H, Nordin S, Lindvall K. Cognitive and emotional reactions to pictorial-based risk communication on subclinical atherosclerosis: a qualitative study within the VIPVIZA trial. *Scand J Prim Health Care* 2023:1-12. doi: https://doi.org/10.1080/02813432.2023.2178850
4. Andersson EM, Lindvall K, Wennberg P, Johansson H, Nordin S. From risk communication about asymptomatic atherosclerosis to cognitive and emotional reactions and lifestyle modification. *BMC Psychol* 2024;**12**:47. doi: https://doi.org/10.1186/s40359-023-01467-x
5. Andersson EM, Liv P, Nordin S, Naslund U, Lindvall K. Does a multi-component intervention including pictorial risk communication about subclinical atherosclerosis improve perceptions of cardiovascular disease risk without deteriorating efficacy beliefs? *Soc Sci Med* 2024;**341**:116530. doi: https://doi.org/10.1016/j.socscimed.2023.116530
6. Holmberg H, Glader EL, Naslund U, Carlberg B, Sonnerstam E, Norberg M, et al. Improved adherence to statin treatment and differences in results between men and women after pictorial risk communication-a sub-study of the VIPVIZA RCT. *Eur J Clin Pharmacol* 2024. doi: https://doi.org/10.1007/s00228-024-03694-6
7. Holmberg H, Sjolander M, Glader EL, Naslund U, Carlberg B, Norberg M, et al. Time to initiation of lipid-lowering drugs for subclinical atherosclerosis: sub-study of VIPVIZA randomized controlled trial, with single-arm cross-over. *Eur Heart J Open* 2022;**2**:oeac003. doi: https://doi.org/10.1093/ehjopen/oeac003
8. Whitmore K, Zhou Z, Magnussen CG, Carrington MJ, Marwick TH. Influence of Repeated Plaque Visualization on Cardiovascular Risk Reduction after 3 years; a randomized controlled trial. *Eur J Prev Cardiol* 2024. doi: https://doi.org/10.1093/eurjpc/zwae026
9. Marwick TH, Whitmore K, Nicholls SJ, Stanton T, Mitchell G, Tonkin A, et al. Rationale and design of a trial to personalize risk assessment in familial coronary artery disease. *Am Heart J* 2018;**199**:22-30. doi: https://doi.org/10.1016/j.ahj.2017.09.011
10. Venkataraman P, Huynh Q, Nicholls SJ, Stanton T, Watts GF, Marwick TH, et al. Impact of a coronary artery calcium-guided statin treatment protocol on cardiovascular risk at 12 months: Results from a pragmatic, randomised controlled trial. *Atherosclerosis* 2021;**334**:57-65. doi: https://doi.org/10.1016/j.atherosclerosis.2021.08.002
11. Coffeng JK, van der Ploeg HP, Castellano JM, Fernandez-Alvira JM, Ibanez B, Garcia-Lunar I, et al. A 30-month worksite-based lifestyle program to promote cardiovascular health in middle-aged bank employees: Design of the TANSNIP-PESA randomized controlled trial. *Am Heart J* 2017;**184**:121-132. doi: https://doi.org/10.1016/j.ahj.2016.11.002
12. Garcia-Lunar I, van der Ploeg HP, Fernandez Alvira JM, van Nassau F, Castellano Vazquez JM, van der Beek AJ, et al. Effects of a comprehensive lifestyle intervention on cardiovascular health: the TANSNIP-PESA trial. *Eur Heart J* 2022;**43**:3732-3745. doi: https://doi.org/10.1093/eurheartj/ehac378
13. Diederichsen AC, Rasmussen LM, Sogaard R, Lambrechtsen J, Steffensen FH, Frost L, et al. The Danish Cardiovascular Screening Trial (DANCAVAS): study protocol for a randomized controlled trial. *Trials* 2015;**16**:554. doi: https://doi.org/10.1186/s13063-015-1082-6

14. Lindholt JS, Sogaard R, Rasmussen LM, Mejldal A, Lambrechtsen J, Steffensen FH, et al. Five-Year Outcomes of the Danish Cardiovascular Screening (DANCAVAS) Trial. *N Engl J Med* 2022;**387**:1385-1394. doi: https://doi.org/10.1056/NEJMoa2208681
15. Vonder M, van der Aalst CM, Vliegenthart R, van Ooijen PMA, Kuijpers D, Gratama JW, et al. Coronary Artery Calcium Imaging in the ROBINSCA Trial: Rationale, Design, and Technical Background. *Acad Radiol* 2018;**25**:118-128. doi: https://doi.org/10.1016/j.acra.2017.07.010
16. van der Aalst CM, Denissen S, Vonder M, Gratama JWC, Adriaansen HJ, Kuijpers D, et al. Screening for cardiovascular disease risk using traditional risk factor assessment or coronary artery calcium scoring: the ROBINSCA trial. *Eur Heart J Cardiovasc Imaging* 2020;**21**:1216-1224. doi: https://doi.org/10.1093/ehjci/jeaa168
17. Denissen SJ, van der Aalst CM, Vonder M, Oudkerk M, de Koning HJ. Impact of a cardiovascular disease risk screening result on preventive behaviour in asymptomatic participants of the ROBINSCA trial. *Eur J Prev Cardiol* 2019:2047487319843396. doi: https://doi.org/10.1177/2047487319843396
18. Denissen S, van der Aalst CM, Vonder M, Gratama JWC, Adriaansen HJ, Kuijpers D, et al. Screening for coronary artery calcium in a high-risk population: the ROBINSCA trial. *Eur J Prev Cardiol* 2021;**28**:1155-1159. doi: https://doi.org/10.1177/2047487320932263
19. Moldovanu D, de Koning HJ, Vonder M, Gratama JWC, Adriaansen HJ, Roeters van Lennep JE, et al. Short-term impact of cardiovascular screening by traditional risk assessment or coronary artery calcium score on health-related quality of life: the ROBINSCA trial. *Eur Heart J Open* 2024;**4**:oeae080. doi: https://doi.org/10.1093/ehjopen/oeae080
20. Norberg M, Wall S, Boman K, Weinehall L. The Västerbotten Intervention Programme. Background, design and implications. *Global Health Action* 2010;**3**:4643. doi: https://doi.org/10.3402/gha.v3i0.4643
21. Piepoli MF, Hoes AW, Agewall S, Albus C, Brotons C, Catapano AL, et al. 2016 European Guidelines on cardiovascular disease prevention in clinical practice: The Sixth Joint Task Force of the European Society of Cardiology and Other Societies on Cardiovascular Disease Prevention in Clinical Practice (constituted by representatives of 10 societies and by invited experts)Developed with the special contribution of the European Association for Cardiovascular Prevention & Rehabilitation (EACPR). *Eur Heart J* 2016;**37**:2315-2381. doi: https://doi.org/10.1093/eurheartj/ehw106

LEGENDS TO SUPPLEMENTARY FIGURES VIPVIZA

Supplementary Figure 1: Crude time trends of Framingham Risk Score in subgroups by sex (A), age-groups (B), educational level (C), and by risk of CVD according to SCORE2 at baseline(D) in both groups from baseline over the one-year and three-year follow-up visits and up to the six-year follow-up.

Supplementary Figure 2: Crude time trends of Systolic Blood pressure in subgroups by sex (A), age-groups (B), educational level (C), and by risk of CVD according to SCORE2 at baseline(D) in both groups from baseline over the one-year and three-year follow-up visits and up to the six-year follow-up.

Supplementary Figure 3: Crude time trends of smoking in subgroups by sex (A), age-groups (B), educational level (C), and by risk of CVD according to SCORE2 at baseline(D) in both groups from baseline over the one-year and three-year follow-up visits and up to the six-year follow-up.

Supplementary Figure 4: Crude time trends of weight in subgroups by sex (A), age-groups (B), educational level (C), and by risk of CVD according to SCORE2 at baseline(D) in both groups from baseline over the one-year and three-year follow-up visits and up to the six-year follow-up.
