# Supplementary material for: Evaluation after delayed and repeated intervention in the VIPVIZA-extended randomized controlled trial: beneficial results 6 years after baseline
Source: Eur Heart J Open. 2026 Apr 13;6(2):oeag047. doi: 10.1093/ehjopen/oeag047 (PMC13075482; doi:10.1093/ehjopen/oeag047)
Supplement: oeag047_Supplementary_Data [file oeag047_supplementary_data.zip › Supplementary Figure 1 VIPVIZA.pdf]

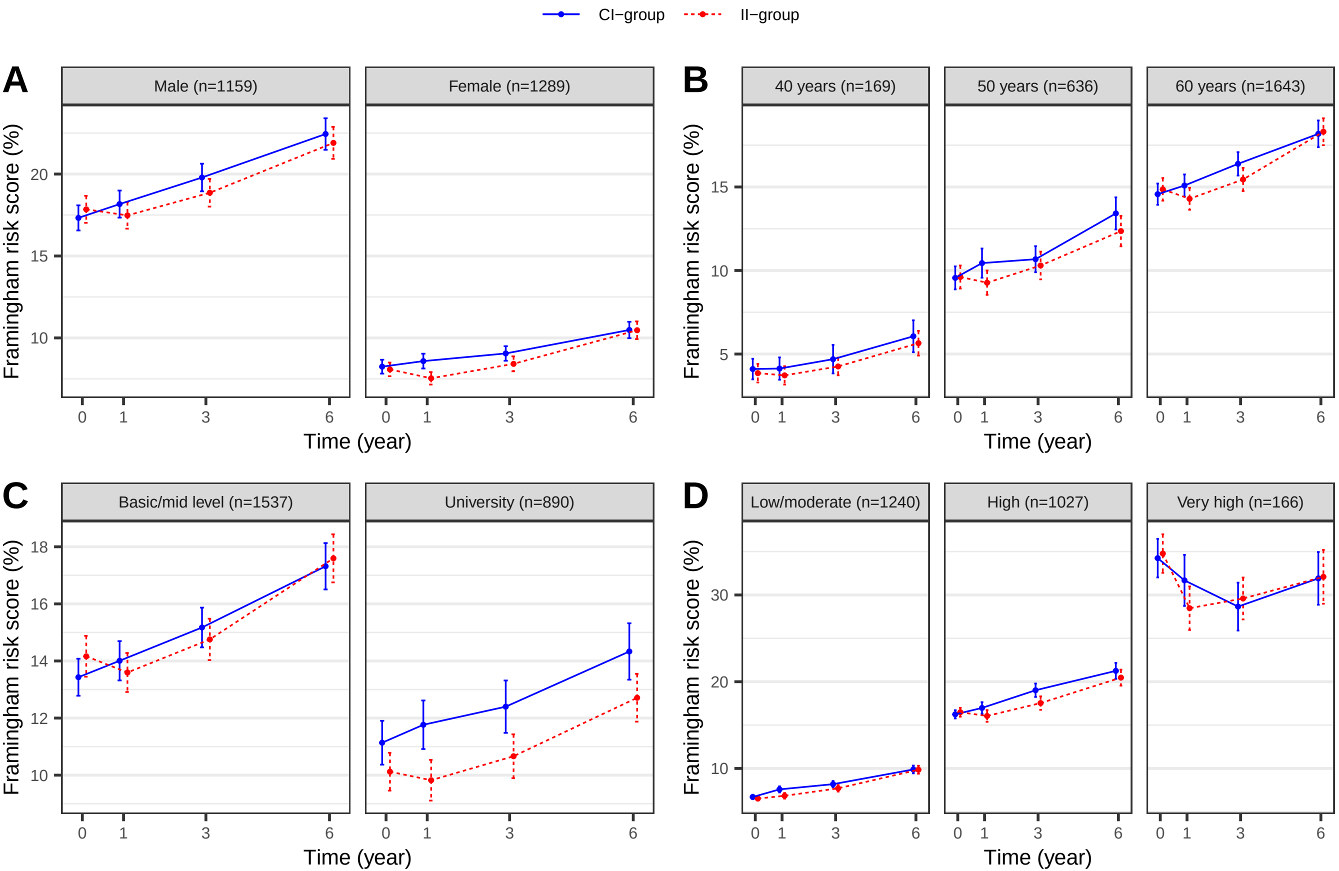

CI−group
II−group
A
Male (n=1159)
Female (n=1289)
B
40 years (n=169)
50 years (n=636)
60 years (n=1643)
C
Basic/mid level (n=1537)
University (n=890)
D
Low/moderate (n=1240)
High (n=1027)
Very high (n=166)
Framingham risk score (%)
Time (year)
