# Supplementary figures and images for: Evaluation after delayed and repeated intervention in the VIPVIZA-extended randomized controlled trial: beneficial results 6 years after baseline
Source: Eur Heart J Open. 2026 Apr 13;6(2):oeag047. doi: 10.1093/ehjopen/oeag047 (PMC13075482; doi:10.1093/ehjopen/oeag047)

—●— CI-group    - - -●- - - II-group

**A**

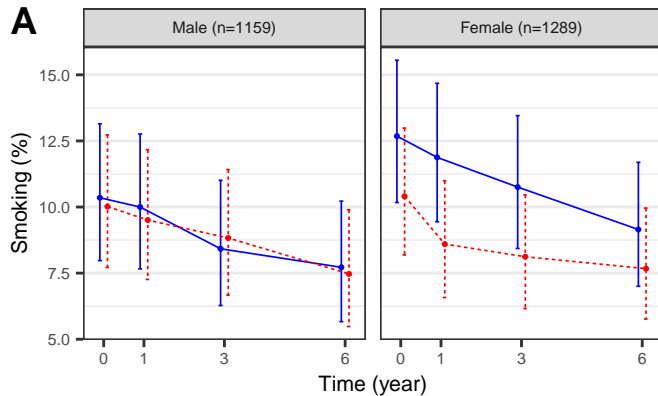

**B**

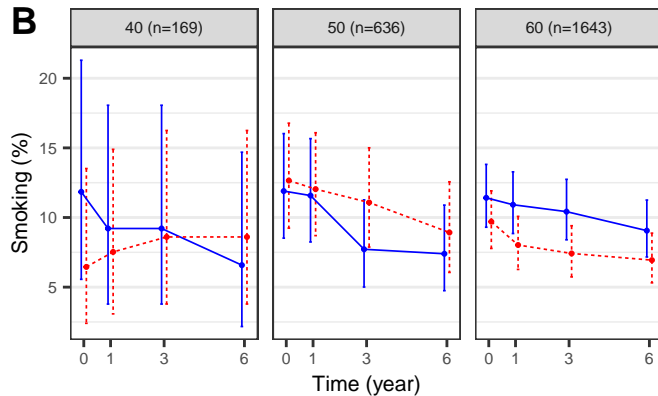

**C**

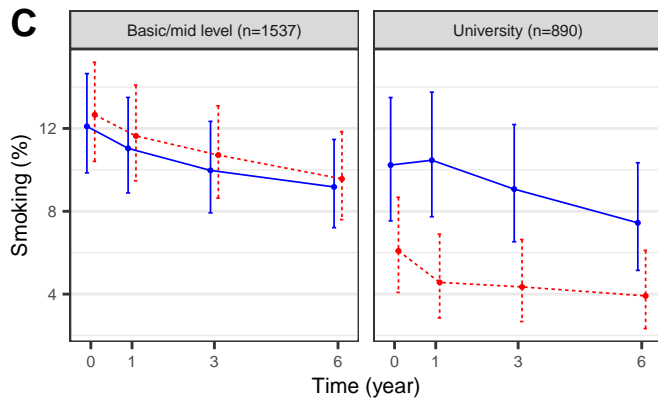

**D**

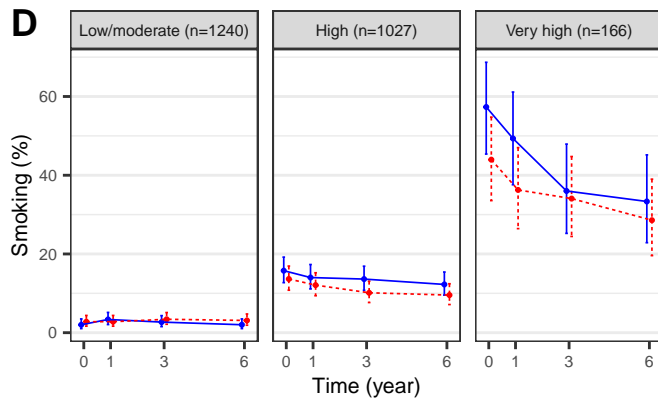

Supplement: oeag047_Supplementary_Data [file oeag047_supplementary_data.zip › Supplementary Figure 3 VIPVIZA.pdf]

—●— CI-group    - - -●- - - II-group

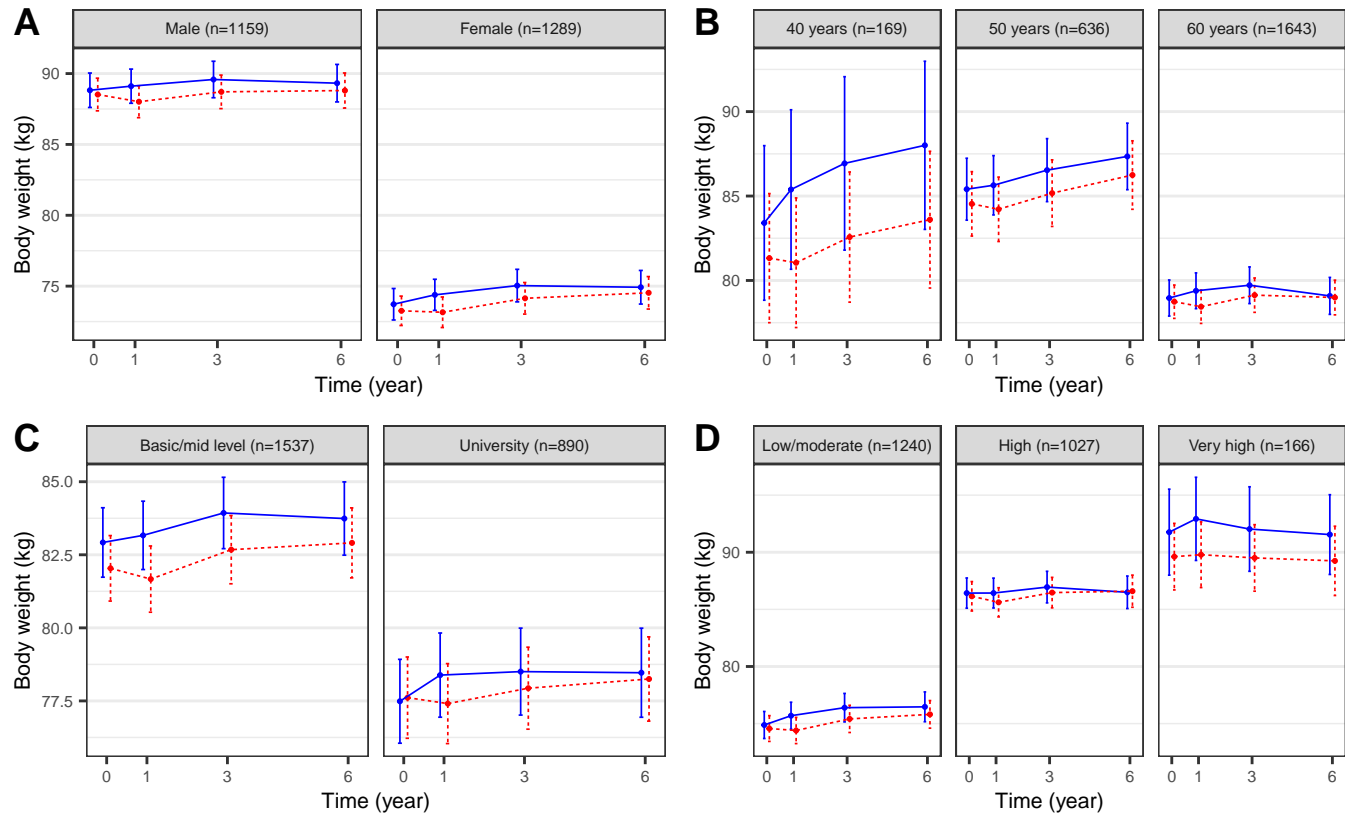

Supplement: oeag047_Supplementary_Data [file oeag047_supplementary_data.zip › Supplementary Figure 4 VIPVIZA.pdf]
